# Neutrophil-to-Lymphocyte Ratio Predicts Sepsis in Adult Patients Meeting Two or More Systemic Inflammatory Response Syndrome Criteria

**DOI:** 10.5811/westjem.18466

**Published:** 2024-06-28

**Authors:** Vamsi Balakrishnan, Anna Yang, Donald Jeanmonod, Harrison Courie, Spencer Thompson, Valerian Peterson, Rebecca Jeanmonod

**Affiliations:** * St. Luke’s University Health Network, Bethlehem, Pennsylvania; † Regional One Health, Memphis, Tennessee; ‡ Linn County Emergency Medicine PC, Cedar Rapids, Iowa; § Medical College of Wisconsin, Department of Emergency Medicine, Grand Rapids, Michigan

## Abstract

**Introduction:**

Determining which patients who meet systemic inflammatory response syndrome (SIRS) criteria have bacterial sepsis is a difficult challenge for emergency physicians. We sought to determine whether the neutrophil-to-lymphocyte ratio (NLR) could be used to exclude bacterial sepsis in adult patients who meet ≥2 SIRS criteria and are being evaluated for sepsis.

**Methods:**

Consenting adult patients meeting ≥2 SIRS criteria and undergoing evaluation for sepsis were enrolled. We recorded patient age, gender, vital signs, and laboratory results. We then later reviewed health records for culture results, end organ dysfunction, survival to discharge, and final diagnoses. Patients were classified as having sepsis if they met ≥2 SIRS criteria and were ultimately diagnosed with a bacterial source. We analyzed data using descriptive statistics and sensitivity and specificity analyses. A receiver operating characteristic curve (ROC) was created to determine test characteristics.

**Results:**

A total of 231 patients had complete datasets. Patients’ median age was 69 (interquartile range [IQR] 54–81), and 49.6% were male. There were 154 patients (66.7%) ultimately diagnosed with sepsis with an identified bacterial source, while 77 patients with ≥2 SIRS criteria had non-infectious reasons for their presentations (33.3%). Septic patients had a median NLR 12.36 (IQR [interquartile range] 7.29–21.69), compared to those without sepsis (median NLR 5.62, IQR 3.89–9.11, *P* < 0.001). The NLR value of 3 applied as a cutoff for sepsis had a sensitivity of 96.8 (95% confidence interval [CI] 92.2–98.8), and a specificity of 18.2 (95% CI 10.6–29.0). The ROC for NLR had an area under the curve of 0.74.

**Conclusion:**

The neutrophil-to-lymphocyte ratio is a sensitive tool to help determine which patients with abnormal SIRS screens have bacterial sepsis.

Population Health Research Capsule What do we already know about this issue?

*Current screening for bacterial sources of sepsis is neither sensitive nor specific.*

What was the research question?

*Can the neutrophil lymphocyte ratio (NLR) be used to exclude bacterial sepsis in patients meeting* ≥ *2 SIRS criteria for systemic inflammatory response syndrome?*

What was the major finding of the study?

*An NLR value of 3 had a sensitivity of 96.8% (CI 92.2–98.8), and a specificity of 18.2% (10.6–29.0). The ROC for NLR had an area under the curve of 0.74.*

How does this improve population health?

*Bacterial sepsis is a major cause of morbidity and mortality. The NLR is a sensitive and inexpensive tool that can help determine which patients have bacterial sepsis.*

## INTRODUCTION

The clinical progression of systemic inflammatory response syndrome (SIRS) from a serious infection to sepsis to septic shock is a major cause of patient morbidity and mortality. Sepsis affects 1.7 million adults in the United States each year, resulting in at least 350,000 deaths.^1^ Worldwide, sepsis is the leading cause of death, passing cardiovascular disease and cancer.^2^

The healthcare costs associated with sepsis are also high, with an estimated annual economic burden of $57 billion in the US in 2019.^3^ Not surprisingly, the cost incurred from any individual case of sepsis increases with increasing disease severity, and disease severity increases over time.^4^ ^,^ ^5^ Therefore, rapid identification and treatment of sepsis has been a high priority within the hospital setting for decades, and performance improvement programs with standardization and protocolization of sepsis management have demonstrated improvement in outcomes.^6^ ^–^ ^8^ Since 87% of cases of sepsis are present upon arrival to the hospital (as opposed to being hospital acquired), emergency physicians play a crucial role in promptly recognizing and intervening with these high-risk patients.^1^ ^,^ ^9^ Emergency physicians are also tasked with limiting unnecessary use of broad-spectrum antibiotics as part of the antibiotic stewardship goals outlined by the Infectious Disease Society of America, the Society for Healthcare Epidemiology of America, and the Pediatric Infectious Disease Society.^10^

Identification of sepsis continues to be a challenge. Screening tools based on clinical and/or laboratory criteria, such as Quick Sequential Organ Failure Assessment (q-SOFA) and SIRS, are commonly used to identify patients with sepsis, but 15–60% of patients who meet those criteria do not go on to have an actual diagnosis of sepsis.^11^ ^–^ ^13^ Cardiac dysrhythmias, primary lung disease, viral illness, trauma, endocrine disease, and numerous other processes can result in a positive SIRS or q-SOFA screen; more importantly, for risk- stratification tools, high sensitivity is critically important to avoid missing cases of sepsis. In multiple trials, SIRS with suspicion for infection outperforms qSOFA in terms of sensitivity but is inferior for specificity.^14^ ^–^ ^18^ Lactate is another tool commonly used primarily for prognostication and assessment of response to treatment in patients with diagnoses of sepsis.^19^ ^,^ ^20^ Although specific lactate values are used as defining criteria in the diagnoses of severe sepsis and septic shock, lactate is neither sensitive nor specific during early sepsis.

The neutrophil-to-lymphocyte ratio (NLR) has been demonstrated to be a reliable and easy-to-obtain measure of patient immune response to a variety of different infectious and non-infectious conditions.^21^ A normal NLR is generally considered <3, although neonatal literature uses more conservative cutoffs.^21^ The NLR has been associated with the presence of bacteremia and appears to be relevant to prognosis and progression in sepsis.^22^ ^–^ ^25^ In neonates, research demonstrates that the NLR can be predictive of both early and late sepsis.^26^ ^–^ ^28^ In this study, we sought to determine whether the NLR could be used to exclude sepsis in adult patients who met two or more SIRS criteria or had a positive q-SOFA screen who were being evaluated for sepsis.

## MATERIALS AND METHODS

### Study Design

This was a prospective cohort study of adult (≥18 years) emergency department (ED) patients who were being evaluated for sepsis. All patients completed written informed consent or had surrogate informed consent. The study was reviewed and approved by the institutional review board.

### Study Setting and Population

The study took place at a single academic ED with an annual census of 50,000 patients. Patients were enrolled from April 2019–March 2020. Adult patients were eligible for enrollment if they were positive on SIRS screening (≥2 values present) or on q-SOFA screening on arrival to the ED and were undergoing evaluation for sepsis. The SIRS criteria are temperature >100.4°F or temperature <96.8°F; heart rate >90 beats per minute; respiratory rate (RR) >20 or pCO_2_ < 32 millimeters of mercury (mm Hg); and white blood cell count >12,000 or <4,000, or band count >10%. A qSOFA score is calculated by giving the patient a point for altered mental status, systolic blood pressure less than 100 mm Hg, or RR ≥22, with ≥2 points considered indicative of high risk. Patients were identified through the electronic health record (EHR) (Epic Systems Corporation, Verona, WA), which generates an automatic computer alert for patients with positive qSOFA and SIRS screens. After identification of potential patients through the computer alert, eligibility forms were completed by clinicians.

Patients were required to provide written informed consent. In cases where patients were too ill, cognitively impaired, or intubated and could not consent, consent was sought from their healthcare proxy, if available. We excluded non-English-speaking patients and patients for whom their evaluations could not be delayed to accommodate the consent process. Pregnant patients and patients triaged to the trauma bay were also excluded. Trauma bay triage criteria are listed in Appendix 1.

### Study Protocol and Measurements

After eligibility forms were completed by clinicians involved in the patients’ care, the forms were screened by study investigators who then approached the patients to obtain consent. Once consent was completed, we recorded clinical data for each SIRS-positive and qSOFA patient enrolled, including age, gender, vital signs, complete blood count (CBC) and lactate levels. We later reviewed health records out to 90 days for culture results; end organ dysfunction (including renal failure, shock liver, pulmonary failure, cardiac failure, delirium); survival to discharge; disposition (home, rehab, nursing facility, or death within 90 days); and final diagnoses. We collected data in a standardized Excel spreadsheet (Microsoft Corp, Redmond, WA) on a password-protected computer belonging to a study investigator. All data was entered from a prepopulated pull-down menu to reduce data entry errors. Once all clinical data collection was complete, the database was purged of patient identifiers prior to analysis.

Patients were classified as having sepsis if they met ≥2 SIRS criteria or 2+ qSOFA criteria and were ultimately diagnosed with a bacterial source. Patients were classified as not having sepsis if they met SIRS/qSOFA criteria but were diagnosed with an alternative source for those abnormalities and had no bacterial source identified. Bacterial sources of sepsis were classified as urinary, pulmonary, central nervous system, intra-abdominal, skin and soft tissue, hematogenous, or other. Cutoff values for analysis of NLR were 3, which is a commonly reported number in the literature as being abnormally high, and 10, which is a value reported to have negative prognostic implications.

### Investigator and Enrollee Training

Only physicians who had completed mandatory CITI training and good clinical practice training consented and enrolled patients in this study. Although all clinicians working in the ED were informed and reminded of the study during weekly educational time and were permitted to complete eligibility forms, only trained investigators were permitted to enroll patients. In addition to mandatory research training, investigators met with the principal investigator (AY) who designed the data collection tool and were trained in its use.

### Data Analysis

We analyzed data using descriptive statistics. Sensitivity and specificity calculations were performed using MedCalc (MedCalc Software Ltd, Ostend, Belgium) and VassarStats (Richard Lowry 1998–2023). We created receiver operating characteristic (ROC) curves to determine test characteristics.

## RESULTS

We enrolled 233 patients. Two had incomplete data sets, leaving 231 patients for data analysis. The patients’ median age was 69, with an interquartile range of 54–81; 49.6% were male. Twenty-five patients (10.7%) were admitted to the intensive care unit, 18 patients (7.7%) died in the hospital during the index visit or were discharged to hospice, and 32 (13.7%) died within 90 days of follow-up. Five patients were enrolled despite not meeting SIRS or qSOFA criteria on initial presentation. They were included in this analysis on an intent-to-treat basis. Further patient characteristics are shown in the Table.

**Table.** Characteristics of patients undergoing evaluation for bacterial source of sepsis.

	Total (N = 231)			
Median age (IQR)	69 (54–81)			
Gender (%)				
Male	114 (49.4)			
Female	117 (50.6)			
Disposition from index visit (%)				
Home	156 (67.5)			
Assisted living and nursing home	55 (23.8)			
Hospice	14 (6.1)			
Death	5 (2.2)			
Unknown	1 (0.4)			
Number of SIRS criteria (%)				
0	0 (0)			
1	6 (2.6)			
2	102 (44.2)			
3	93 (40.3)			
4	26 (11.3)			
5	4 (1.7)			
Number of qSOFA criteria (%)				
0	94 (40.7)			
1	113 (48.9)			
2	23 (10)			
3	1 (0.4)			

*IQR*, interquartile range; *SIRS*, systemic inflammatory response syndrome; *qSOFA*, quick Sequential Organ Failure Assessment.

**Figure 1.**
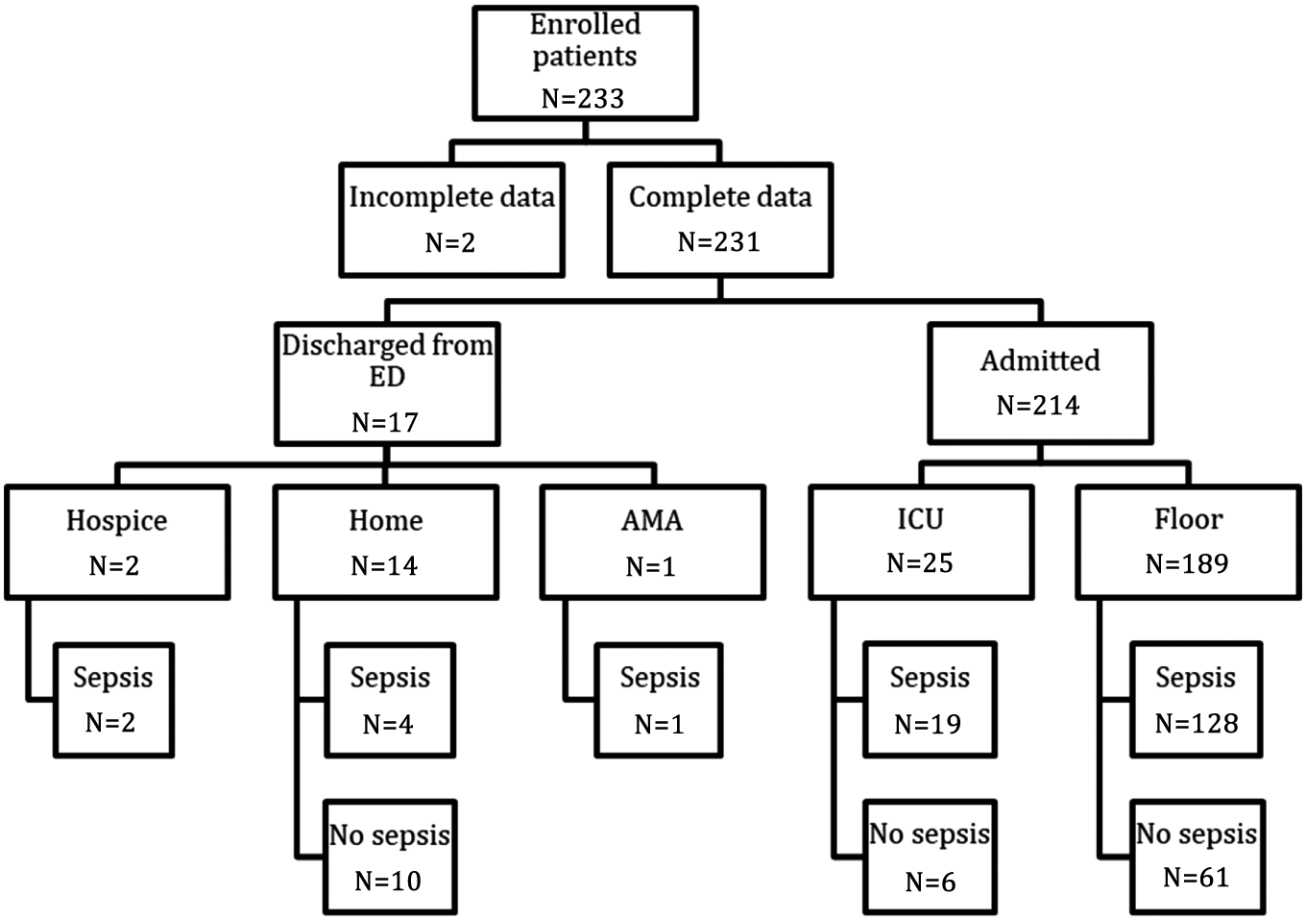
Patient flow diagram. *ED*, emergency department; *AMA*, against medical advice; *ICU*, intensive care unit.

**Figure 2.**
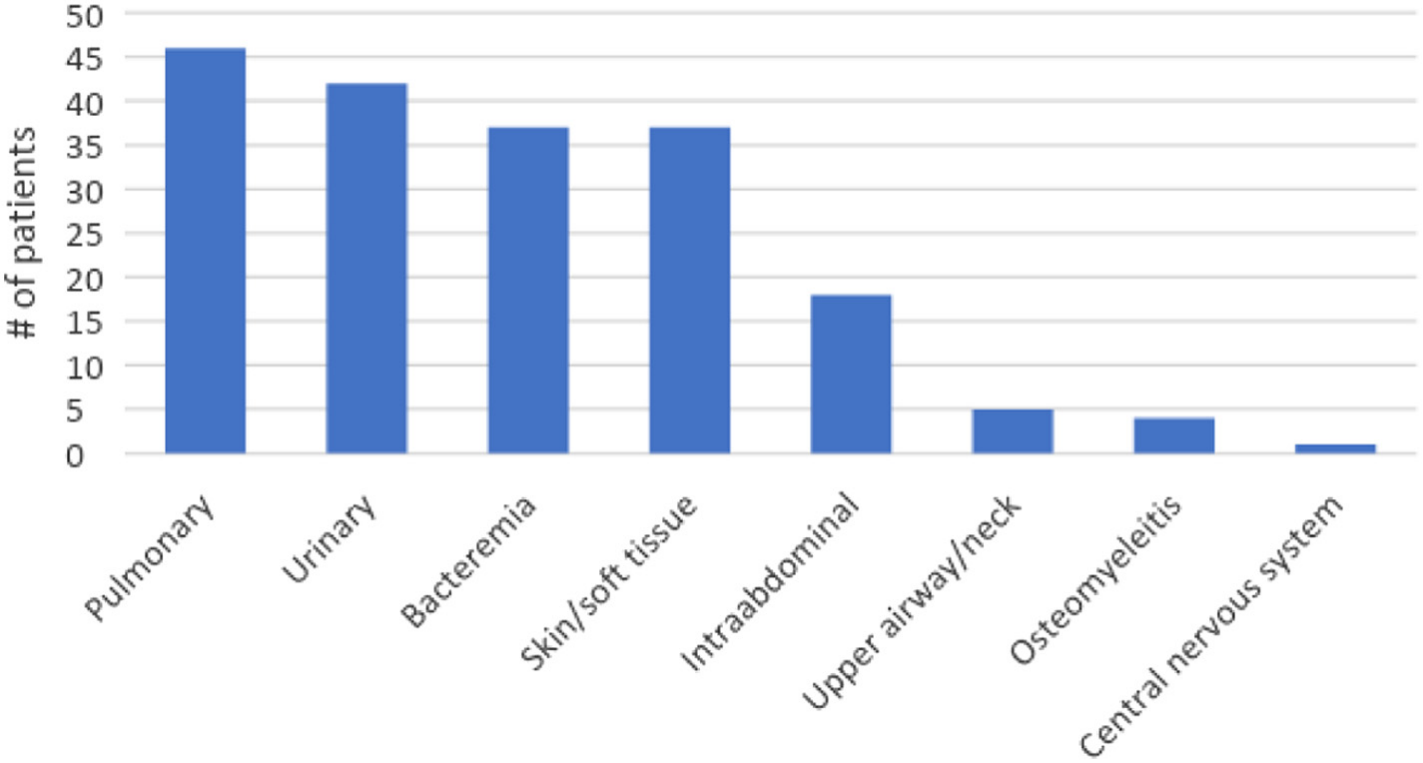
Sources of sepsis.

**Figure 3.**
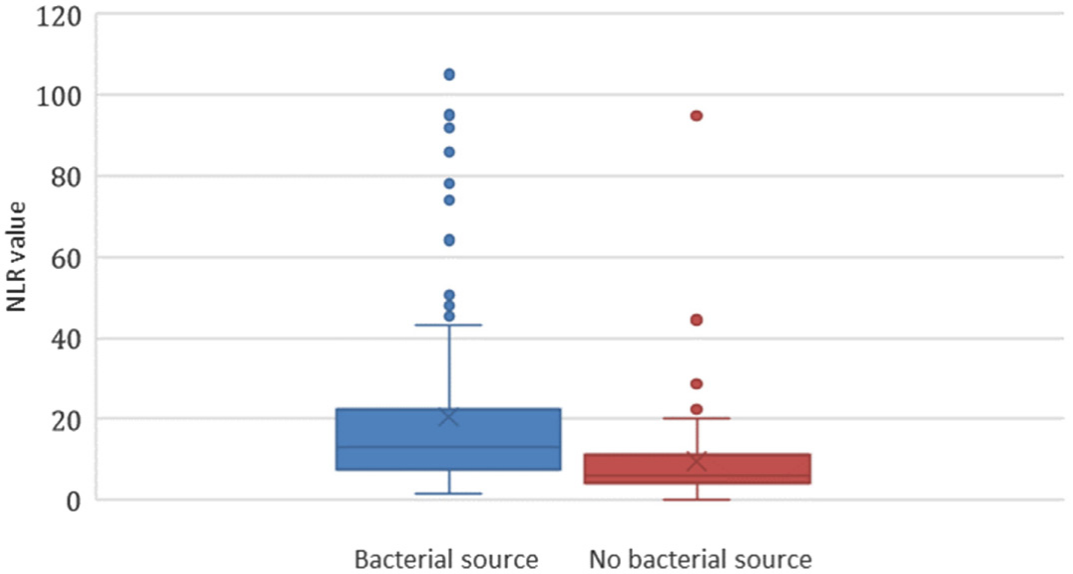
Neutrophil-to-lymphocyte ratio in patients with ≥2 SIRS* criteria. *SIRS, systemic inflammatory response syndrome; *NLR*, neutrophil-to-lymphocyte ratio.

Of the 231 eligible patients, 154 patients (66.7%) were ultimately diagnosed with sepsis with an identified bacterial source (Figure 1). Seventy-seven had non-infectious sources identified (33.3%). The most commonly identified sources were pulmonary and urinary tract, followed by bacteremia and soft tissue infections (Figure 2). Some patients had more than one identified source. The most commonly identified reasons for patients meeting SIRS criteria without bacterial source of sepsis were viral syndromes (28 patients); congestive heart failure (10); asthma/chronic obstructive pulmonary disease exacerbations (5); medication/drugs (5); malignancy related (5); pneumonitis (5); and complications of endocrine disease (3). Patients with sepsis with an identified bacterial source had a median NLR of 12.36 (interquartile range [IQR] 7.29–21.69), compared to a median NLR of 5.62 (IQR 3.89–9.11) in those that did not have a bacterial source (*P* < 0.001) (Figure 3).

**Figure 4.**
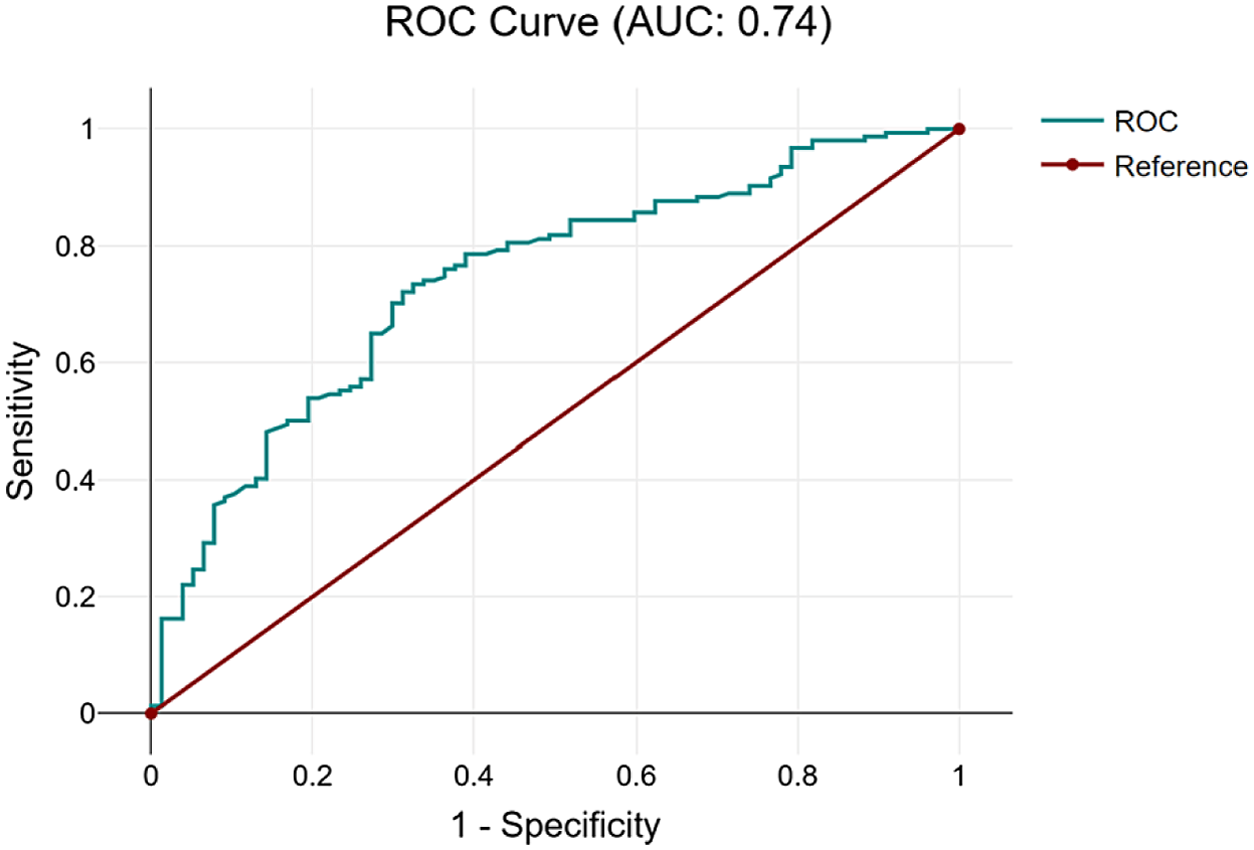
Receiver operating curve for test characteristics of neutrophil-to-lymphocyte ratio in predicting bacterial source in patients meeting ≥2 SIRS* criteria. *ROC*, receiver operator curve; *SIRS, systemic inflammatory response syndrome.

The NLR value of 3 applied as a cutoff for sepsis had a sensitivity of 96.8 (95% confidence interval [CI] 92.2–98.8), and a specificity of 18.2 (CI 10.6–29.0). In this population with a high prevalence of disease, the positive predictive value of NLR was 70.3 (CI 63.6–76.2), with a negative predictive value of 73.7 (CI 48.6–89.9), with a performance odds ratio of 6.86 (CI 2.37–19.89) for having disease. When an NLR cutoff of 10 was used, the specificity increased significantly to 73%, although with a marked sacrifice of sensitivity down to 60.5%. The ROC for NLR yielded an area under the curve of 0.74 (Figure 4).

## DISCUSSION

Most hospitals use some variation of screening for sepsis as required to meet core sepsis metrics implemented by the Centers for Medicare and Medicaid Services. These are typically based on SIRS and/or qSOFA criteria with suspicion for infection. Many hospitals have best practice advisory warnings automatically implemented into the EHR based on these stratification tools to help clinicians earlier identify potentially ill patients who may benefit from escalation of care. This initial recognition of a higher risk cohort of patients is an important first step. Because these initial screens are historically insensitive (both with sensitivities of about 50%) but also not adequately specific, a secondary screen would be useful to help determine which patients might have sepsis as the reason for their presentation.^13^ ^,^ ^29^ A NLR is a simple calculation requiring only a complete blood count (CBC) with differential to be resulted and could be used as a secondary screen.

Although virtually every patient with suspected sepsis has their CBC analyzed, the goal of this blood test is traditionally to determine absolute white blood cell count and percentage of bands, as these are existing criteria within SIRS. The NLR has not been posited as being useful in the determination to initiate antibiotics or as a potential screen for sepsis. For a computer-generated alert, using a NLR is easy to implement and could provide useful guidance in prescreened high-risk patients.

In our study, patients—all of whom were prospectively being evaluated for sepsis—a NLR >3 gave an odds ratio of 6.9 of sepsis, with a sensitivity of 96.8%. This suggests that it may be reasonable to initiate broad-spectrum antibiotics in high-risk patients with an elevated NLR. Since a CBC has a rapid lab turnaround time, this may hasten treatment in patients for whom the physician is “on the fence” regarding antibiotic administration.

Whether NLR would be useful in acute presentations of sepsis with negative SIRS and qSOFA screens is difficult to assess. We chose to include only patients with positive clinical exam features for sepsis, which represents workup bias within our study. This was done so that we could have a standardized protocol by which to enroll patients, rather than simply enrolling every patient who had a CBC drawn. There are many clinical presentations in which patients “screen negative” for sepsis using qSOFA and SIRS criteria, but the physician has a high index of suspicion for other reasons that are less easily quantified, or in whom sepsis is still on the differential diagnosis. These “screen negative” patients with sepsis are by definition the hardest to recognize, and NLR may have the potential to risk-stratify them. Further study may help to identify the role that NLR could have in screening patients.

It is important to acknowledge the performance of qSOFA and SIRS within the context of our study. Very few of our enrolled patients who went on to have a diagnoses of sepsis with bacterial sources had positive qSOFA screens, which is not in keeping with the existing literature on qSOFA scores. Only 11% of our bacterial sepsis patients had positive qSOFA scores, and 29% of our positive qSOFA screens were not septic. Because of this, it is difficult to speak to NLR’s benefit in patients with positive qSOFA screens, as this represented the minority of our patient population, most of whom were enrolled on the basis of vital sign abnormalities consistent with SIRS. This would be an area for further study.

## LIMITATIONS

Because this was a relatively small study performed at a single institution and we enrolled patients as a convenience sample, its generalizability is limited. The incidence of disease was very high in our cohort, which is important to consider when interpreting our results. We enrolled a relatively high-risk cohort, which means that our data may not be applicable to patients with negative qSOFA/SIRS screens. To effectively mirror real-world application of the NLR in patients, we attempted to identify patients in a prospective manner to include patients with physiologic processes that cause vital sign abnormalities where infection might have been a consideration in the differential diagnosis, but ultimately the diagnosis was not sepsis. Further, this study is also limited by the study protocol inclusion criteria. Since this was a prospective study with written consent and patients with altered mental status could not consent, and surrogate consent was not always available, this could have affected the yield of NLR as a screening tool, either positively or negatively.

Since our study required qSOFA and SIRS screening for study entry, it is limited by the accuracy with which those screens were performed. At our institution, these values are input by a triage nurse and may be subject to individual variability and skillset. Examples of how this could have affected the study include inaccuracies in temperature measurements based on method or inaccuracies in counting respiratory rates. Additionally, our qSOFA data likely was affected by the lack of emphasis on it; historically, and currently, our institution stresses the importance of consideration for SIRS with suspicion of source of infection, with qSOFA of secondary import. It is possible that we may not have emphasized the documentation of it as much as SIRS given somewhat less familiarity, resulting in missed opportunities for enrollment in patients who were SIRS negative but qSOFA positive.

Additional limitations include the lack of inclusion of patients who were excluded from enrollment by study design. Non-English speakers were excluded because of inability to appropriately translate the informed consent for all comers. Although the drawing of blood is of minimal risk to the fetus, pregnant patients were excluded from the current study because during pregnancy the neutrophil count naturally increases during the second and third trimesters. Additionally, physiologic changes of pregnancy including increased heart rate and respiratory rate might make the patient SIRS positive, despite the absence of a severe infection. Patients triaged to the trauma bay were excluded because of difficulties with consenting patients and dictating diagnostic testing during their trauma evaluation.

This study was performed in a period prior to the COVID-19 pandemic. Because of issues with quarantining patients early in the pandemic, patient enrollment was stopped at the beginning of the pandemic. The utility of NLR as a screening test may not be applicable in a post-pandemic environment.

## CONCLUSION

The neutrophil-to-lymphocyte ratio is a sensitive tool to help identify patients who may have a bacterial source for sepsis. It is fast to use and without additional cost to the patient. In our study, acceptable performance was demonstrated with a cutoff of 3. Further studies should focus on validation of these findings in broader populations.

## Supplementary Information



