# Supplementary material for: Neutrophil-to-Lymphocyte Ratio Predicts Sepsis in Adult Patients Meeting Two or More Systemic Inflammatory Response Syndrome Criteria
Source: West J Emerg Med. 2024 Jun 28;25(5):690–6. doi: 10.5811/westjem.18466 (PMC11418870; doi:10.5811/westjem.18466)
Supplement: Supplementary file 1 [file wjem-25-690-s001.docx]

Appendix 1: Trauma bay triage criteria

| \| GCS criteria \| GCS ≤ 13, GCS motor score $\leq$ 5 or drop in GCS by 2 from baseline \| \| --- \| --- \| \| Vital sign criteria \| Systolic blood pressure < 90  Respiratory rate < 10 or > 29 breaths per minute  Intubated \| \| Anatomic injuries \| Penetrating injuries to head, torso or extremities proximal to the elbow and knee  Fracture of 2 or more long bones (humerus and femur)  Amputations proximal to the wrist and ankle  Crushed, degloved, or pulseless injured extremities  Blunt abdominal injury with firm or distended abdomen  Suspected pelvic fractures  Suspected open or depressed skull fractures  Paralysis from suspected spinal cord injury \| \| Mechanism \| Falls of > 20 feet or 3 times the height of a child, falls with head strike on anticoagulants or clopidogrel (but not aspirin)  High risk motor vehicle accidents (intrusion greater than 12 inches, ejection, or death in the same passenger compartment)  Bicycle and motorcycle accidents > 20 miles per hour  Pedestrians or bicyclists struck at > 20 miles per hour  Drownings  Partial thickness or full thickness burns of > 10% body surface area  Major trauma involving pregnant women of > 20 weeks gestation \| |
| --- | --- | --- | --- | --- | --- | --- | --- | --- |
